# Influence of Recurrent Laryngeal Nerve Transient Unilateral Palsy on Objective Voice Parameters and on Voice Handicap Index after Total Thyroidectomy (Including Thyroid Carcinoma)

**DOI:** 10.3390/ijerph18084300

**Published:** 2021-04-18

**Authors:** Zuzana Veldova, Richard Holy, Jan Rotnagl, Temoore Younus, Jiri Hlozek, Jaromir Astl

**Affiliations:** 1 Department of Otorhinolaryngology and Maxillofacial Surgery, Military University Hospital, 16902 Prague, Czech Republic; zuzana.veldova@uvn.cz (Z.V.); jan.rotnagl@uvn.cz (J.R.); temoore.younus@lf3.cuni.cz (T.Y.); jiri.hlozek@uvn.cz (J.H.); jaromir.astl@seznam.cz (J.A.); 2 Third Faculty of Medicine, Charles University, 10000 Prague, Czech Republic

**Keywords:** total thyroidectomy, recurrent laryngeal nerve paresis, Voice Handicap Index, speech range profile

## Abstract

*Introduction:* Total thyroidectomy (TT) is one of the most common surgical endocrine surgeries. Voice impairment after TT can occur not only in patients with recurrent laryngeal nerve (RLN) transient paralysis, but also in cases of normal vocal cord mobility. *Aim:* To compare voice limits using a speech range profile (SRP) in patients before and 14 days after TT and to investigate the influence of the early results of voice quality after TT on the personal lives of patients. We focused on the perception of voice change before and shortly after TT. *Materials and methods:* A retrospective study, in the period 2018–2020, included 65 patients aged 22–75 years. We compared two groups of patients: group I (*n* = 45) (without RLN paresis) and group II (*n* = 20) (with early transient postoperative RLN paresis). Patients underwent video flexible laryngocopy, SRP, and Voice Handicap Index-30 (VHI-30). *Results:* In group I, the mean values of F_max_ (maximum frequency) and I_max_ (maximum intensity) decreased in women (both *p* = 0.001), and VHI-30 increased (*p* = 0.001). In group II after TT in women, the mean F_max_ and I_max_ values decreased (*p* = 0.005 and *p* = 0.034), and the frequency range of the voice was reduced from 5 to 2 semitones. The dynamic range of the voice was reduced by 3.4 dB in women and 5.1 dB in men.VHI-30 increased (*p* = 0.001). *Conclusion:* The study documented a worsening of the mean values of SRP, VHI-30, and voice parameters of patients in group II. Voice disorders also occurred in group I without RLN paresis. Non-paretic causes can also contribute to voice damage after TT. SRP and VHI-30 are suitable tools for comparing voice status in two groups of patients, including those with dysphonia. Our data support the claim that the diagnosis of a thyroid cancer does not necessarily imply a higher postoperative risk of impaired voice quality for the patient.

## 1. Introduction

Voice changes after total thyroidectomy (TT) often have a neurogenic cause and are one of the most common complications after thyroid surgery. TT is indicated for the treatment of autoimmune inflammatory disorders (thyrotoxicosis), endemic goiter, and malignant and benign tumors. The change in voice in patients after TT can be caused by

- nerve injury during TT;
- paretic (or plegic) causes of voice damage: recurrent laryngeal nerve (RLN) paresis or superior laryngeal nerve (SLN) paresis;
- a non-paretic cause (e.g., laryngopharyngeal reflux disease (LPRD)).

Only a thorough videolaryngostroboscopy examination performed in the preoperative period can reveal the above causes of dysphonia. A patient’s vocal cords must be examined before each TT. These must be differentiated from potential laryngeal nerve injury-RLN paresis after TT [1]. Hoarseness caused by RLN paresis is one of the most common postoperative complications of TT after hypocalcaemia [2]. Consequences resulting from nerve injury voice deterioration after TT (postoperative RLN paralysis) differs depending on whether the injury of RLN corresponds to neurapraxia (nerve compression, which primarily damages the myelin sheath and secondarily the axons), axonotmesis (traction injury by excessive nerve strain followed by axon fracture) or neurotmesis (complete or partial nerve break) according to Seddon’s Classification [3,4,5] and whether the damage or injury is unilateral or bilateral. 

The incidence of postoperative RLN paresis ranges from 0–8% in primary thyroid surgery. It is used as a criterion to measure the success of surgical treatment [6].

The identification of RLN injuries in terms of transient paresis in 0.7% of patients, and persistent paresis in 0.2% of patients, is reported by some authors [7]. 

RLN injury during thyroidectomy/parathyroidectomy occurs intraoperatively significantly more frequently in visually intact RLN than in the transected nerves [8]. The forward motor branch of RLN bifurcation near the Berry ligament is particularly at risk of traction injury. This is made evident by a deterioration in voice quality after TT (higher average jitter) in an effort to maintain the radicality of surgery (Berry ligament) in the cases of malignant thyroid tumors [6]. TT is a standard treatment for thyroid tumors including carcinomas [7,9].

Voice change after TT is reported in the literature in 16–89% of cases [10,11], but is often not considered to be associated with neurogenic impairment (RLN paresis) [10,12]. 

Suitable questionnaires for the subjective evaluation of VHI voice quality are reported in the literature [10,13,14]. In practice, the VHI-10 and VHI-30 questionnaires are used as an evaluation of voice quality as a parameter of postoperative quality of life.

There is an Italian study of voice professionals (teachers) that shows the benefits of the VoiSS VTDS questionnaires for a preventive voice program for teachers [15].

The non-paretic causes of voice change after TT include

- aero-digestive disorders: proximal acid reflux–loss of coordination of upper esophageal sphincter (UES) [10];
- perioperative trauma of extralaryngeal muscles [11,16];
- modified vascular supply and venous drainage-postoperative soft tissue edema, and mucosal edema-postintubation (orotracheal) [9];
- local neck pain (psychological responses after surgery) [12];
- changes in laryngeal mucosa;
- neurogenic (psychogenic) causes.

Patients without RLN paresis may have problems with voice quality, early voice fatigue, and limited voice range. Regardless of the etiology of voice disorders after TT, voice change is a feared complication for patients, especially vocal professionals (opera singers, pop music singers, actors, teachers, speakers, managers, translators, members of choirs, actresses, lawyers, doctors). That is why follow-up phoniatric care is so important for all patients who have undergone TT. Logopedic therapy in patients with post-thyroidectomy dysphonia improves the vocal and postural outcomes [14].

## 2. Materials and Methods

Our monocentric study included 65 patients indicated for TT. All patients were examined between January 2018 and September 2020. The group consisted of 57 women and 8 men, and the mean age in the group was 45 years (range 22–75 years).

All operations were performed by senior surgeons at one institution. 

During all TT operations intraoperative neuro-monitoring (IONM) was used. 

In the case of IONM, short- or medium-acting muscle relaxants were used on administration of anesthesia. Invasive needle sensing electrodes were applied to the m. vocalis. Non-invasive electrodes present on the oro-tracheal tube were placed between the voice ligaments under the control of direct laryngoscopy. 

A monopolar stimulation electrode was used (stimulus intensity 0.5–1 mA). We evaluated action potential (AP) with an amplitude of more than 100 µV with adequate latency from the stimulation site as an adequate functional nerve response. 

Electro-myographic recording was performed on a MEDTRONIC instrument NIM-NEURO 3.0. A true-positive result (TP) occurred when the IONM detected RLN palsy, which was confirmed postoperatively.

A false positive was considered to be a condition where, despite the IONM anticipated RLN paresis, the vocal cords were postoperatively moving. The true negative result was a condition where the IONM assumed that the RLN functionality was maintained, which was confirmed postoperatively. False negative results were evaluated as a condition where, despite the IONM detecting preserved function of RLN, postoperative paresis occurred.

To reduce the incidence of false-negative and false-positive responses, we monitored neural structures according to the scheme: vagus nerve before dissection, recurrent laryngeal nerve before dissection, RLN after dissection, vagus nerve after dissection. 

All patients included in the study (with intraoperative IONM obtained in a non-physiological/pathological response or postoperatively caused by changing voice) were promptly investigated (objective voice parameters and VHI-30 questionnaire) within 14 days after TT.

The inclusion criteria were as follows:

- age over 18 years;
- preoperative normal laryngeal finding;
- preoperatively normal voice;
- non-physiological/pathological response obtained perioperatively at IONM;
- postoperative change of voice.

The exclusion criteria were as follows:

- age below 18 years;
- history of any neurological disorders or diseases (e.g., multiple sclerosis);
- any preoperative benign vocal cord lesions or other voice disorders;
- any preoperative pathological videolaryngoscopic findings;
- hearing loss requiring hearing aids.

We divided the patients after TT into two groups:

- GROUP I with movable vocal cords (verified by laryngostroboscopic examination) (without RLN paresis) *n* = 45;
- GROUP II patients with postoperative unilateral transient RLN paresis *n* = 20.

The indications for TT surgery were as follows: nodular goiter *n* = 25 (38%), thyroiditis with thyreotoxicosis *n* = 10 (15%), diffuse goiter *n* = 7 (11%), Graves–Basedow goiter n = 6 (9%), multinodular goiter *n* = 6 (9%), papillary thyroid carcinoma (papillary carcinoma, papillary variant of papillary carcinoma, foliculary variant of papillary carcinoma) *n* = 6 (9%), Hashimoto´s autoimmune thyroiditis *n* = 3 (5%), medullary thyroid carcinoma *n* = 1 (2%), and retrosternal nodular goiter *n* = 1 (2%). 

All patients underwent the following examinations before TT and check-up within 14 days after TT.

Performed examinations:

- Optical methods: video flexible laryngoscopy;
- Acoustic methods: jitter (%);
- Examination of voice field of conversational voice (determination of basic average voice position Fo (Hz) and basic sound pressure level SPL (dB (A));
- Aerodynamic examination: maximum phonation time (MPT) (s);
- Psychometric examination: VHI-30 (Voice Handicap Index);
- Speech range profile (SRP) (dynamic and frequency range of speaking voice) loud reading and reading with low voice intensity not whisper.

We used the LingWAVES software system for voice analysis.

We analyzed the periodicity of the voice using the perturbation parameter (jitter) and examined the voice field of the speech voice (determination of the average mean voice position Fo (Hz) and the basic sound pressure level SPL (dB (A))), SRP (speech range profile) using silent and loud reading methods to determine the lowest frequency (F_min_ Hz), highest frequency (F_max_ Hz), minimum intensity (I_min_ dB SPL), and maximum intensity (I_max_ dB SPL). We evaluated the changes in the glottal gap using an aerodynamic test using the prolonged phonation of the isolated vowel (A), thus measuring the maximum phonation time. All sound recordings were made at a distance of 30 cm from the microphone in a quiet room with an ambient noise level of less than 40 dB (A)). When recording standard text readings, the normal volumes of the conversational voice (habitual reading), silent voice (semi-voice), and loud reading were used.

Psychometric examinations were evaluated by completing the VHI-30 (Voice Handicap Index) questionnaires.

Methodology: All patients signed an informed consent for total thyroidectomy before the surgeries. Our study was followed according to the Declaration of Helsinki. Due to the retrospective type and character of the study, approval by the local ethics committee was not required.

## 3. Results

The total percentage of transient RLN paresis was 31%. It was always a transient RLN paresis. 

From the point of view of thyroid histology, transient unilateral paresis was most often found in group II in patients with nodular goiter, *n* = 8 (40%), and thyreotoxicosis, *n* = 5 (25%).

Laterality of transient RLN Paresis in Group II:

In terms of laterality, transient RLN paresis prevailed on the left side: 60% (*N* = 12). Right-sided transient RLN paresis was 40% (*N* = 8). 

Results of objective voice parameters: 

In Group I, only 2 parameters changed significantly after the TT: jitter increase (*p* = 0.001), and VHI-30 point increase (*p* = 0.001).

Fo values in women (habitual voice-reading standard text with medium voice intensity) were statistically significantly lower than before the operation (*p* = 0.016).

The values of Fo in men did not differ statistically significantly after TT; there was only a slight deepening of the voice (decrease in the pitch). 

In Group II after the TT, all observed parameters changed significantly (worsened): MPT shortening (*p* = 0.001), SPL decrease (*p* = 0.030), jitter increase (*p* = 0.001), and VHI-30 point increase (*p* = 0.001).

The values of Fo (habitual voice-reading standard text with medium voice intensity) in women did not change significantly after TT (*p* = 0.836).

The values of Fo in men differed only on the significance level *p* = 0.1 (not statistically significant); we noticed a slight increase in voice position and an increase in voice pitch.

Test speech range profile (SRP) (the most important parameter) results are shown in see Table 1.

SRP Group I:

Basic statistical characteristics of F_min_/_max_ and I_min_/_max_ and the results of the non-parametric Wilcoxon test between before and after TT (before and after):

Women: the values for loud reading differed statistically significant: for F_max_, voice height decreased after surgery (*p* = 0.001). But I_max_ also decreased after surgery, there was a limitatiton of load reading ability (*p* = 0.001). 

Men: none of the monitored parameters proved to be statistically significantly different between F_max_ and I_max_ after TTE. The pitch of the voice or the intensity of the voice in the upper part of the voice field (VF) were almost unchanged. 

SRP Group II:

Basic statistical characteristics of F_min_/_max_ and I_min_/_max_ and the results of the non-parametric Wilcoxon test before and after TTE: 

Women: after TT, the VF (voice field) parameters of the loud reading F_max_ and I_max_, were significantly different (*p* = 0.005) and (*p* = 0.034). Here, too, there was a reduction in voice height while trying to read loudly, thus limiting the ability to amplify the voice. 

Men: F_max_ values did not change after TT; only I_max_ differed, but only at a significance level of 0.1 (*p* = 0.068). Thus, the ability to read aloud in men with RLNp after TT was not significantly reduced.

Results—voice field speaking voice SRP (see Table 2):

Group I Women: The voice frequency range from the original 5 semitones (G3-C4) was limited to 4 semitones (G3-H3) in the upper part of the voice field. Dynamic voice range reduced from 15.1 dB SPL (dB (A)) to 13.0 dB SPL (dB (A)) mainly at higher voice field frequencies (limiting voice intensity during loud reading). 

Men: After the TT, the lower part of the voice field (VF) was freely expanded and the upper part of the VF in loud reading remained unchanged. There was a change in the extent of VF before surgery 5 semitones (C3-F3) versus after surgery 8 semitones (A2-F3).

The dynamic range of the voice increased at loud reading, at the same frequency level as before TT, i.e., 12.3 dB (SPL (A)) before and 13.6 dB (SPL (A)) after TT. 

Group II Women: We noticed the restriction in the voice field in the frequency and dynamic range, in both parts of the voice field and in the lower and upper parts. 

The voice pitch was limited in both the low and high frequencies. Frequency range decreased from 5 semitones (F3#-H3) to 2 semitones (G3-A3#); voice dynamics decreased from 14.2 dB SPL (dB (A)) to 10.7 dB SPL (dB (A)). 

Men: Shifting voice pitch to deeper parts of the frequency spectrum, limited ability to increase voice pitch and intensity from loud reading, from original 4 semitones (A2#-D3#) to 3 semitones (A2#-C3#), dynamic range reduced from 19.0 dB SPL (dB (A)) to 13.9 dB SPL (dB (A)). 

Results of VHI-30 questionnaires (see Table 3):

The total score significantly changed (worsened) in Group II (*p* = 0.001), but also in Group I (*p* = 0.001). 

Our results: We noticed mild voice problems when evaluating the each parts of the VHI-30 (Physical, Functional, Emotional parts), VHI-30 Total—we noticed no patient deteriorated by more than 18 points. Overview in Table 3. 

Patients with thyroid Carcinoma: 

5 patients with papillary carcinoma were without paresis postoperatively.

1 patient with papillary carcinoma and 1 patient with medullary carcinoma had transient unilateral RLN paresis.

In the group of 5 patients with thyroid Carcinoma without postoperative RLN paresis, there was a reduction in FR by an average of 1 semitone. VHI-30 total points increased by only 5.5 points. The dynamic range of the voice decreased after TTE by 4 dB SPL (dB (A)), by 0.64 dB SPL (dB (A)) more than the average in the group of all patients without postoperative RLN paresis.

1 patient with papillary carcinoma had transient unilateral RLN paresis. After TTE, the VHI-30 increased from 6 to 70 points. The dynamic range of the voice after TTE decreased by 13 dB SPL (dB (A)).

1 patient with medullary carcinoma had transient unilateral RLN paresis. After TTE, the VHI-30 had not changed before 4/after 4 points. The dynamic range of the voice after TTE decreased by only 1.3 dB SPL (dB (A)). This patient had not been shown to have NEM2A or B, Hirschsprung’s disease.

## 4. Discussion

The results of our study indicate, in agreement with the world literature, the importance of two parameters in monitoring voice analysis in patients after TT [17]: SRP (speech range profile) and VHI-30. Previously monitored parameters such as jitter and shimmer are losing importance in voice quality assessment today.

Our results support the idea that the SRP is an important indicator of the change in voice after TT. Our data show a difference of Fo max–Fo min in women in group II (with RLN paresis) during loud reading and semi-voice reading, limiting the frequency range of the voice from the original 5 semitones (F3#-H3) to 2 semitones (G3-A3), especially in the higher frequency range. The finding correlates, among other things, with the finding of glottis insufficiency due to unilateral RLN paresis after TT. In contrast, Bihari et al. found in their study that there was no change in tone range in patients with unilateral RLN paresis [18]. 

Reduction of the frequency range of the voice in women of Group II in our study, of 3 semitones in both the lower and upper parts of the frequency spectrum, may be due to both paretic (RLNparesis, EBSLNp) and non-paretic causes of voice disorders (most often proximal acid reflux or mucosal-orotracheal postintubation edema).

Another part of the SRP rating is Dynamic Voice Range and it has a similar predicative value. Our results show that Group II women with RLN paresis were able to use the same low voice intensity (I_min_) after TT as before surgery, which was measured during reading by semi-voice.

According to some authors such as Ma et al. and Siupsinskiene et al. [19,20], the lower the I_min_, the lower the subglottic pressure. The I_min_ parameter is a basic predictor of voice disorder, which is one of the basic indicators of improving the dynamic range of the voice during rehabilitation. 

According to Leino et al., in men, an enormous increase in I _max_ is a sign of voice fatigue, and at the same time a decrease in the dynamic range of the voice signals a voice disorder [21].

In our study in Group I (without RLN), paradoxically, after the TT, the dynamic range of the speaking voice was expanded. 

In Group II (with RLN paresis), however, as expected, the Dynamic Voice Range was reduced by 5.1 dB SPL (dB (A)). 

Our study shows that I _max_ is not, in contrast to Leino et al. [21], a significant indicator of voice disorders in men (after TT). With regard to changes in the dynamic range of voice (decrease) our results are similar to Leino et al. [21].

D´Alatri with Marchese showed in their research the importance of the SRP. 

They compared SRP and VRP (Voice Range Profile) in healthy subjects and dysphonic patients [22]. 

In common phoniatric practice, VRP (singing examination) is performed only on vocal professionals. VRP has no application in patients with dysphonia that has occurred after TT. The SRP is a useful alternative tool for assessing voice limits [22].

The importance of VHI-30-evaluation of individual parts of VHI-30 questionnaires after TT shows an increase in the number of points (deterioration) in the physical, functional, and emotional parts. In both of our evaluated groups, the most dissatisfaction with voice was manifested in the physical part, and the least in the emotional part.

Our results correlate with Dehqan et al. [23], where the authors reported in their group of patients with unilateral RLN paresis a direct correlation between VHI-30 total and the physical part of VHI-30 [23]. In Group I (without RLN transient paresis), a worsening of the total VHI-30 score was found in 31% of patients, whereas in Group II (with transient RLN paresis), the worsening of the total number was 75%.

In our study, in Groups I and II we noticed a direct correlation between the subjective assessment of patient voice quality and with the results of their voice analysis parameters and SRP (frequency and dynamic range of voice). Patients with the greatest SRP restriction and impaired perturbation parameters were more dissatisfied with their voice and vice versa. 

Although these included patients without RLN paresis, they had worse VHI-30 scores. This fulfills our assumptions that non-paretic causes of voice changes (alongside paretic causes) and predictive factors contribute to the resulting patient satisfaction with their voice and to their application in personal and professional life. 

The results of the SRP and VHI-30 examinations are relevant for the future, as they contribute to the development of new rehabilitation procedures, which will enable patients to use their voice without restrictions in communication processes in everyday life.

## 5. Conclusions

The study shows that in the case of monitoring changes in voice after TT, the most important objective voice parameters are SRP and VHI-30. These are very sensitive indicators of voice changes that can be used both in patients with dysphonia (after TT) caused by transient RLN paresis and in patients with non-paretic dysphonia.

Our data support the claim that the diagnosis of thyroid cancer does not necessarily imply a higher postoperative risk of impaired voice quality for the patient.

## Figures and Tables

**Table 1:** SRP: mean values, standard deviations, statistical significance.

Parameter	*n*	Before	After	*p*
Group I women				
F_min_ (Hz)	41	195.45 ± 70.22	194.59 ± 74.22	0.588
F_max_ (Hz)	41	256.09 ± 84.29	242.96 ± 87.62	0.001
I_min_ (dB (A))	41	59.64 ± 19.66	59.64 ± 19.30	0.423
I_max_ (dB (A))	41	74.74 ± 22.59	72.60 ± 21.59	0.001
Group I men				
F _min_ (Hz)	4	127.35 ± 42.60	111.13 ± 18.03	0.655
F_max_ (Hz)	4	177.75 ± 60.16	172.25 ± 64.69	0.655
I_min_ (dB (A))	4	56.23 ± 3.93	57.13 ± 2.54	0.285
I_max_ (dB (A))	4	68.55 ± 4.51	70.73 ± 5.55	0.285
Group II women				
F_min_ (Hz)	16	190.27 ± 24.21	193.43 ± 22.08	0.328
F_max_ (Hz)	16	249.57 ± 31.49	228.26 ± 18.73	0.005
I_min_ (dB (A))	16	54.54 ± 2.74	54.41 ± 3.71	0.477
I_max_ (dB (A))	16	68.74 ± 3.99	65.17 ± 6.59	0.034
Group II men				
F_min_ (Hz)	4	118.93 ± 35.25	118.48 ± 31.33	1.000
F_max_ (Hz)	4	153.45 ± 36.70	140.85 ± 38.95	0.109
I_min_ (dB (A))	4	51.58 ± 4.45	51.08 ± 1.65	1.000
I_max_ (dB (A))	4	70.60 ± 2.37	64.95 ± 3.31	0.068

SRP = speech range profile; F_min_ = minimum frequency; F_max_ = maximum fraquency; I_min_ = minimum intensity; I_max_ = maximum intensity.

**Table 2:** Voice field of the speaking voice.

Female	Group I	Group II
FR (Hz) before TT (Hz) _(max-min)_	60.7 (G3-C4) 5 st	59.3 (F3#-H3) 5 st
FR (Hz) after TT (Hz) _(max-min)_	48.2 (G3-H3) 4 st	34.8 (G3-A3) 2 st
DR dB SPL (dB (A)) before TT _(max-min)_	15.1	14.2
DR dB SPL (dB (A)) after TT _(max-min)_	13.0	10.8
Difference FR (before/after TT) Difference DR (before/after TT)	−1 st −2.1 dB (SPL(A))	−3 st −3.4 dB (SPL(A))
Male	Group I	Group II
FR (Hz) before TT _(max-min)_	50.4 (C3-F3) 5 st	35.5 (A2#-D3#) 5 st
FR (Hz) after TT _(max-min)_	61.1 (A2-F3) 8 st	22.4 (A2-C3#) 4 st
DR dB SPL (dB (A)) before TT _(max-min)_	12.3	19.0
DR dB SPL (dB (A)) after TT _(max-min)_	13.6	13.9
Difference FR (before/after TT) Difference DR (before/after TT)	+3 st +1.3 dB (SPL(A))	−1 st −5.1 dB (SPL(A))

FR = frequency range; DR = dynamic range; F_min_ = minimum frequency; F_max_ = maximum frequency; I_min_ = minimum intensity; I_max_ = maximum intensity; st = number of semitones; # = semitone higher than previous full tone.

**Table 3:** Voice Handicap Index (VHI) VHI–30.

	Group I *n* = 45		Group II *n* = 20	
Part of the VHI-30	Before TT Average Value	After TT Average Value	Before TT Average Value	After TT Average Value
Physical	0.91	3.7	0.85	7.9
Functional	0.37	2.3	1.0	6.3
Emotional	0.24	1.7	0.4	3.7
VHI-Total (SD)	1.6 (2.99)	6.93 (5.95)	2.2 (2.42)	18.4 (19.22)

SD = Standard Deviation.

## Data Availability

Not applicable.
